# Problematic internet use and shyness among Chinese adolescents: a moderated mediation model

**DOI:** 10.3389/fpsyg.2024.1439692

**Published:** 2024-08-29

**Authors:** Hong Sun, Yang Yu

**Affiliations:** College of Teacher Education, Taishan University, Tai'an, China

**Keywords:** problematic internet use, self-regulation, social comparison orientation, shyness, adolescents

## Abstract

The potential adverse effects of problematic internet use (PIU) on individuals’ offline personalities have been a significant concern in internet psychology. This study aimed to investigate the negative effects of PIU on shyness and the mediating role of self-regulation, as well as the moderating role of social comparison orientation. A total of 1,322 adolescents from China participated in the survey. The results indicated that: (1) PIU positively predicted shyness; (2) self-regulation partially mediated the relationship between PIU and shyness; (3) social comparison orientation moderated the mediation effect, specifically buffering the negative prediction of PIU on self-regulation, but exacerbating the negative predictive effect of self-regulation on shyness. The findings explored and verified the psychosocial effects of the internet, contributing to a deeper understanding of how PIU affects individual personality traits and offering insights into its role in broader social behavior patterns.

## 1 Introduction

In the current digital age, the widespread use of the internet has made problematic internet use (PIU) an increasingly prominent social issue. PIU is a behavioral pattern characterized by a tendency toward unreasonable internet use, leading to negative consequences in daily life (Spada, 2014). Researchers now prefer the term “problematic use” over “addiction” or “dependence” because it is broader and more inclusive, covering issues such as internet addiction and tendencies among adolescents (Lavoie et al., 2023). Although numerous studies have highlighted the negative impacts of PIU (Romero-López et al., 2021; Asam et al., 2019), there is limited exploration of PIU’s predictive role on individual personality and its internal mechanisms. Orchard and Fullwood (2010) suggested that internet usage behavior reflects both online behavior traits and offline personality characteristics, indicating that PIU can predict and assess individual personality traits. Aboujaoude (2017) further noted that internet use might negatively impact offline personalities by fostering impulsivity and amplifying narcissistic traits. Therefore, PIU as unrestrained internet usage behavior may significantly influence individual personality traits.

Shyness, as a personality trait, is typically manifested as withdrawal and avoidance behavior in social or evaluative situations, accompanied by emotions such as tension, anxiety, and embarrassment (Cheek and Buss, 1981). Although early research (e.g., Kagan et al., 1988) focused primarily on the genetic and biological foundations of shyness, recent research have revealed that environmental and behavioral factors also play a crucial role in the formation and development of shyness (Volbrecht and Goldsmith, 2010; Macgowan and Schmidt, 2020). According to Bandura’s theory of Triadic Reciprocal Determinism, there is a complex interplay between individual’s behavior, personal factors, and environment. Therefore, in the context of the widespread use of the internet today, PIU as a significant behavioral pattern may exacerbate shyness traits, leading individuals to become more withdrawn and anxious in face-to-face social interactions. Adolescents, at a critical stage of personality formation and social skill development, are particularly susceptible to PIU due to their high receptivity to new technologies, making them a high-risk group (Bu et al., 2021). This study aims to explore the association between PIU and adolescent shyness, identifying the mediating factors and conditions under which this association is moderated by other variables. The goal is to provide scientific evidence for developing social adaptability intervention programs for adolescents with PIU.

### 1.1 PIU and shyness

First, Social Skills Deficits Theory posited that the lack of necessary social skills was a key factor leading to social anxiety and shyness (Levitan and Nardi, 2009). PIU caused adolescents to spend a significant amount of time in the virtual world, reducing face-to-face social opportunities in reality. They lacked opportunities to practice real-life social skills such as eye contact, interpreting body language, and receiving immediate emotional feedback. This lack of practice opportunities made it difficult for adolescents to acquire necessary social skills (Hutchinson et al., 2004), thereby limiting their social development and contributing to the formation and development of shyness personality traits.

Furthermore, PIU may lead adolescents to form a “safe zone” online, making them feel more comfortable and relaxed in the virtual world. However, this sense of security existed only in the virtual world. When they needed to engage in real-life social interactions, they felt more uneasy due to the lack of online protection. This undoubtedly increased their risk of relying on the internet to satisfy their social needs (Jeong et al., 2020). Long-term internet dependence made it harder for them to adapt to and face real-life social challenges, further exacerbating the development of shyness. Thus, adolescents were caught in a dual dilemma of PIU and shyness.

Empirical research supported this view, showing that while online communication can satisfy certain psychological needs, excessive reliance on online social interactions may lead to a decline in real-life social skills, thereby increasing shyness (Li et al., 2014). Specifically, PIU was shown to significantly predict individual shyness levels (Ebeling-Witte et al., 2007). This study proposed Hypothesis 1: Adolescent PIU positively predicts shyness levels.

### 1.2 The mediating role of self-regulation

Self-regulation referred to an individual’s ability to control attention allocation, emotions, and interpersonal interactions (Fries et al., 2008). On the one hand, PIU consumed a significant amount of cognitive, emotional, and time resources, making it difficult for adolescents to effectively allocate resources and make rational decisions, thus reducing their self-regulation ability (Tomska et al., 2022; Cerniglia et al., 2017). Additionally, individuals with PIU often found it difficult to delay gratification; they tended to seek immediate satisfaction, ignoring long-term goals and plans (Cheng et al., 2020). This behavior pattern further weakened their self-regulation ability, making it more challenging for them to manage time and control impulses. From a neuroimaging perspective, studies provided evidence that individuals with PIU exhibited significant changes in the activity and structure of the prefrontal cortex, which is responsible for impulse control, self-regulation, and decision-making (Hong et al., 2013). This impairment made it difficult for adolescents to self-regulate when facing impulses and desires.

On the other hand, the relationship between self-regulation ability and shyness has been explored in several studies. For example, an early study found that self-regulation ability was an important predictor of an individual’s performance in social situations. Individuals with poor self-regulation were more likely to exhibit emotional outbursts during social interactions, which made them feel more anxious and tense in social settings, leading to higher levels of shyness (Eisenberg et al., 1995). Additionally, individuals with lower self-regulation ability were more likely to exhibit an overactive behavioral inhibition system (BIS), which refers to a heightened sensitivity to potential threats and social evaluation, making them feel more nervous and uneasy in social situations (Ran et al., 2018; Markarian et al., 2013). This increased nervousness and unease can further contribute to higher levels of shyness. Conversely, higher self-regulation ability helps individuals better manage their attention in social situations, avoiding excessive self-focus (Alm, 2007), which can reduce the likelihood of shyness developing.

In summary, self-regulation may be the mediating process through which PIU predicted shyness in adolescents, suggesting that self-regulation mediates the relationship between PIU and shyness (Hypothesis 2).

### 1.3 The moderating role of social comparison orientation

While PIU may predict shyness through both direct and indirect pathways, it is undeniable that these effects may vary among individuals. Therefore, examining whether the relationship between PIU and shyness is moderated by other factors is essential, as it helps answer the question, “When does PIU take effect?”

Social comparison orientation reflected how individuals position themselves in social environments and responded to external evaluations and expectations. It influenced self-perception, emotional states, and how individuals process external information and cope with social pressure (Gibbons and Buunk, 1999). Adolescents were actively exploring self-identity and shaping their sense of self, making social comparison orientation particularly important during this developmental stage (Krayer et al., 2008). This study examined whether individual differences in social comparison orientation affect the relationship between PIU and shyness.

First, according to Social Cognitive Theory (Bandura, 1991), individuals can significantly influence their behavior choices and self-efficacy by observing the actions of others and the consequences of those actions. Social comparison was not only a means of assessing one’s status but also a way to enhance one’s opportunities by learning and mimicking the effective behavior strategies of others (Suls et al., 2002). By observing the behaviors and outcomes of others, individuals could form beliefs about their own abilities, thereby influencing their behavior and emotional regulation capabilities (Zell and Alicke, 2009). Social Comparison Theory (Festinger, 1954) further emphasized that in the absence of objective standards, individuals often evaluated their abilities and achievements by comparing themselves to others. Adolescents with a high social comparison orientation were typically more focused on comparing their status and image with others and might take appropriate actions to maintain or enhance these social evaluation metrics (Bosch et al., 2010; Suls et al., 2002), including making efforts to control their behavior and emotions to better meet social expectations. For adolescents with PIU, this strong motivation for comparison drove them to better control their behavior and emotions to meet social expectations, thus effectively understanding and managing their behavior and its potential consequences. As McIntyre and Eisenstadt (2011) pointed out, social comparison could serve as a self-regulation tool, helping adolescents measure the gap between their actual self and ideal self, ought self, feared self. This meant that although adolescents with PIU often face significant self-regulation challenges, a higher social comparison orientation can help mitigate the negative impact of this issue on self-regulation. Therefore, this study hypothesized that social comparison orientation can buffer the negative predictive effect of PIU on self-regulation (Hypothesis 3).

Secondly, although a high social comparison orientation helped adolescents with PIU better control their behavior and emotions, this comparison can also exacerbate their sensitivity to external evaluations (Luo et al., 2015). This excessive sensitivity can lead adolescents to overinterpret others’ reactions in social situations, thereby increasing their levels of social anxiety and shyness. Specifically, when adolescents with PIU use social comparison as a tool for self-regulation, they may continually focus on comparing their performance in social interactions with others. This constant comparison and fear of negative evaluation can make them behave more reserved and shy in social situations. For example, research found that individuals with a high social comparison orientation often exhibited lower self-esteem and stronger negative emotions after making social comparisons, which made them behave more shyly in social situations (Vogel et al., 2015; Henderson, 2002). Additionally, according to Self-Awareness Theory (Duval and Wicklund, 1972), highly self-monitoring individuals may overly criticize their own behavior during social interactions, leading to feelings of shyness or embarrassment. Therefore, a high social comparison orientation may exacerbate the negative predictive effect of self-regulation on shyness (Hypothesis 4).

In summary, social comparison orientation may play different roles in different contexts. In the relationship between PIU and self-regulation, a high social comparison orientation may help mitigate the negative impact of PIU on self-regulation. However, in the relationship between self-regulation and shyness, a high social comparison orientation might exacerbate shyness levels. Therefore, the social comparison orientation plays dual roles of buffering and exacerbating in these two relationships.

**Figure 1:**
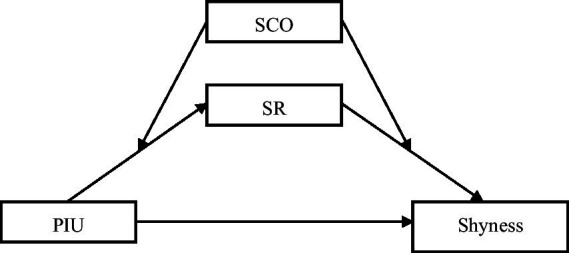
Conceptual model. PIU, problematic internet use; SR, self-regulation; SCO, social comparison orientation.

The research framework for this study is delineated in Figure 1.

## 2 Materials and methods

### 2.1 Participants and procedures

In October 2023, using random cluster sampling, students from two middle schools in Shandong Province, China, were selected. A total of 1,400 questionnaires were distributed, with 1,322, valid responses received, resulting in an 94.4% response rate. The participants included 654 males and 668 females; 637 from rural areas and 685 from urban areas; 580 only children and 742 non-only children. The age range was 12–16 years (*M* = 13.89, SD = 1.42). Ethical approval was obtained from the Ethics Committee of Tai Shan University, and informed consent was secured from students, teachers, and parents prior to conducting the survey. Trained researchers conducted the survey following standardized procedures. The questionnaires were completed in quiet classrooms, taking approximately 20 min. All responses were anonymous.

### 2.2 Measures

#### 2.2.1 Internet addiction test

This study utilized the Chinese version of the Internet addiction Test to measure PIU (Young, 2009). The test consists of 20 items and uses a 5-point Likert scale. Total scores of 0–30 indicate no PIU, 31–49 indicate mild PIU, 50–79 indicate moderate PIU, and 80–100 indicate severe PIU. An example item is, “Do you stay online late into the night instead of sleeping?” The test has high internal consistency and test–retest reliability after meta-analysis reliability is combined (Liu, 2020) and has been widely applied among Chinese college and middle school students (Yu et al., 2019; Liu et al., 2024). In this study, the Cronbach’s *α* was 0.90.

#### 2.2.2 Self-regulation questionnaire

The Chinese Self-Regulation Questionnaire (SRQ), consisting of 38 items, was used to assess self-regulation abilities. Responses were recorded on a 4-point Likert scale from 1 (“strongly disagree”) to 4 (“strongly agree”). Higher scores indicate greater self-regulation. An example item is “When I notice myself daydreaming in class, I make an effort to focus.” The SRQ has been validated in Chinese students (Sun et al., 2024). In this study, the Cronbach’s *α* was 0.91.

#### 2.2.3 Social comparison orientation measure

We used the Chinese version of the Iowa–Netherlands Comparison Orientation Measure, developed by Gibbons and Buunk (1999) and adapted by Wang et al. (2006), to assess social comparison orientation. The 11-item scale includes statements such as “I am always highly attentive to the differences in how I and others approach tasks” rated on a 5-point scale. Higher scores indicate a stronger social comparison orientation. This measure is validated for Chinese students, showing good validity and reliability (Zhang et al., 2023). In this study, the Cronbach’s *α* was 0.71.

#### 2.2.4 Shyness scale

Shyness was assessed using the Revised Cheek and Buss Shyness Scale (RCBS; Cheek, 1983). This 13-item scale includes statements such as “I feel nervous when talking to authoritative people.” and has been widely used among Chinese students (Chen et al., 2021; Yu et al., 2019). Responses are rated on a 5-point scale from 1 (“not at all”) to 5 (“very”), with higher scores indicating greater shyness. In this study, Cronbach’s *α* was 0.74.

### 2.3 Statistical analysis

Data analysis was conducted using SPSS 26.0, along with Hayes’ PROCESS 4.0 macro for SPSS (Hayes, 2013) to perform descriptive statistics, mediation, and moderation tests. All quantitative variables were standardized before analysis. Mediation and moderated mediation effects were assessed using 5,000 bootstrap samples with a 95% confidence interval. Harman’s single-factor test indicated no significant common method bias, as the variance explained by the first factor was only 17.74%, well below the 40% threshold. An exploratory factor analysis conducted on items related to PIU, self-regulation, social comparison orientation and shyness confirmed these findings.

## 3 Results

### 3.1 Preliminary analyses

Table 1 presented the descriptive statistics and correlations of the main variables. PIU was positively correlated with shyness and negatively correlated with self-regulation. Self-regulation was positively correlated with social comparison orientation and negatively correlated with shyness.

**Table 1:** Descriptive statistics and correlations between variables (*N* = 1,322).

	Variables	*M*	SD	1	2	3	4
1	PIU	38.22	13.70	1.000			
2	SR	95.81	13.89	−0.508^**^	1.000		
3	SCO	33.70	6.13	0.103^**^	0.075^**^	1.000	
4	Shyness	34.41	8.33	0.187^**^	−0.260^**^	0.115^**^	1.000

^*^p < 0.05 (two-tailed). ^**^p < 0.01 (two-tailed). PIU, problematic internet use; SR, self-regulation; SCO, social comparison orientation.

The prevalence of mild PIU among adolescents was 44.33% (586/1,322), moderate PIU was 19.44% (257/1,322), and severe PIU was 0.76% (10/1,322).

### 3.2 Testing the mediation model

Mediation tests were conducted using PROCESS Model 4. The results revealed that PIU significantly predicted shyness (*β* = 0.187, *t* = 6.929, *p* < 0.001, 95% CI = [0.134, 0.241]), thus supporting Hypothesis 1. Furthermore, PIU had a significant negative predictive effect on self-regulation (*β* = −0.508, *t* = −21.444, *p* < 0.001, 95% CI = [−0.555, −0.462]), and self-regulation also negatively predicted shyness (*β* = −0.222, *t* = −7.214, *p* < 0.001, 95% CI = [−0.283, −0.162]). Self-regulation partially mediated the relationship between PIU and shyness (*ab* = 0.113, SE = 0.018, 95% CI = [0.078, 0.148]), with the mediation effect accounting for 60.43% of the total effect, thus supporting Hypothesis 2.

### 3.3 Testing the moderated mediation model

A moderated mediation model was examined using PROCESS Model 58. The results, as shown in Table 2, indicated that in the first half of the pathway, PIU had a significant negative predictive effect on self-regulation. Additionally, the interaction between PIU and social comparison orientation significantly predicted self-regulation (*β* = 0.056, *t* = 2.553, *p* < 0.05, 95% CI = [0.013, 0.099]), thus supporting Hypothesis 3. In the second half of the pathway, self-regulation significantly negatively predicted shyness, and the interaction between self-regulation and social comparison orientation significantly predicted shyness (*β* = −0.080, *t* = −3.499, *p* < 0.001, 95% CI = [−0.124, −0.035]), thus supporting Hypothesis 4. Social comparison orientation moderated both the first and second halves of the pathway where PIU predicted shyness.

**Table 2:** Conditional process analysis (*N* = 1,322).

	*β*	SE	*t*	95% CI
**Mediator variable model predicting SR**				
PIU	−0.528	0.024	−22.313^**^	[−0.574, −0.482]
SCO	0.126	0.024	5.336^**^	[0.080, 0.173]
PIU × SCO	0.056	0.022	2.553^*^	[0.013, 0.099]
**Dependent variable model predicting shyness**				
PIU	0.044	0.031	1.431	[−0.016, 0.105]
SR	−0.257	0.031	−8.291^**^	[−0.318, −0.196]
SCO	0.130	0.027	4.853^**^	[0.077, 0.182]
SR × SCO	−0.080	0.023	−3.499^**^	[−0.124, −0.035]
**Conditional effect**	Effect	Boot SE	Boot LLCI	Boot ULCI
*M* – 1SD	0.104	0.024	0.058	0.152
*M*	0.136	0.019	0.100	0.175
*M* + 1SD	0.159	0.026	0.110	0.212

^*^p < 0.05 (two-tailed). ^**^p < 0.01 (two-tailed). Bootstrap sample size = 5,000. PIU, problematic internet use; SR, self-regulation; SCO, social comparison orientation; CI, confidence interval; LL, low limit; UL, upper limit.

**Figure 2:**
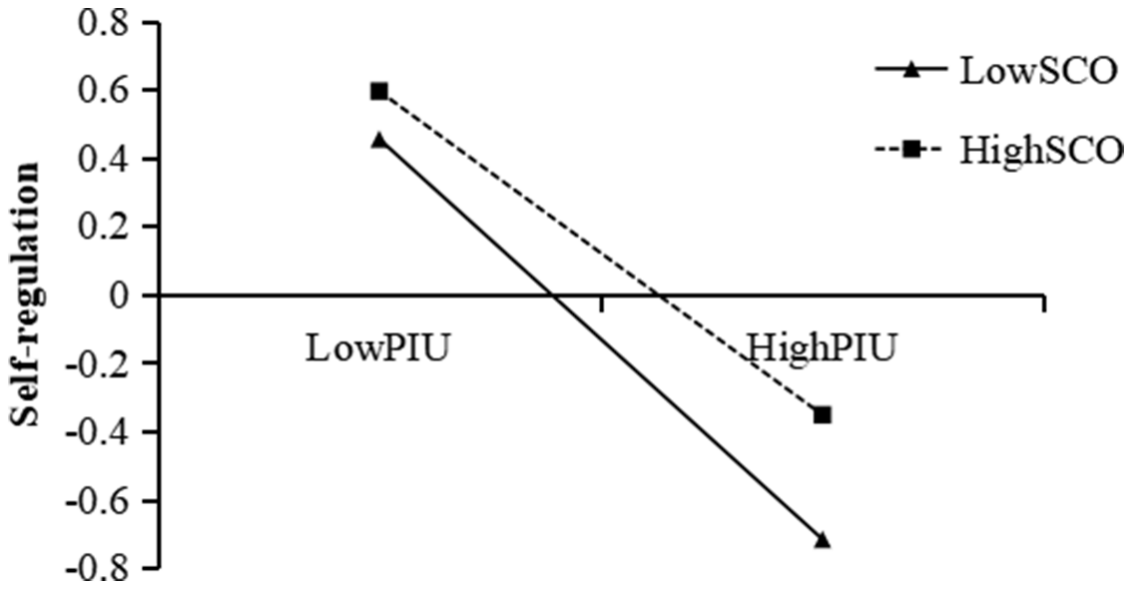
SCO moderated the relationship between PIU and SR. PIU, problematic internet use; SR, self-regulation; SCO, social comparison orientation.

A simple slope test was conducted to examine the interaction effect of PIU and social comparison orientation on self-regulation. The effect values of PIU on self-regulation were calculated when social comparison orientation was at the mean ± 1SD, and a simple effect analysis graph was plotted. As shown in Figure 2, the negative correlation between PIU and self-regulation was significantly lower in adolescents with high social comparison orientation (*β* = −0.472, *t* = −15.535, 95% CI = [−0.532, −0.413]) compared to those with low social comparison orientation (*β* = −0.584, *t* = −17.233, 95% CI = [−0.650, −0.517]).

**Figure 3:**
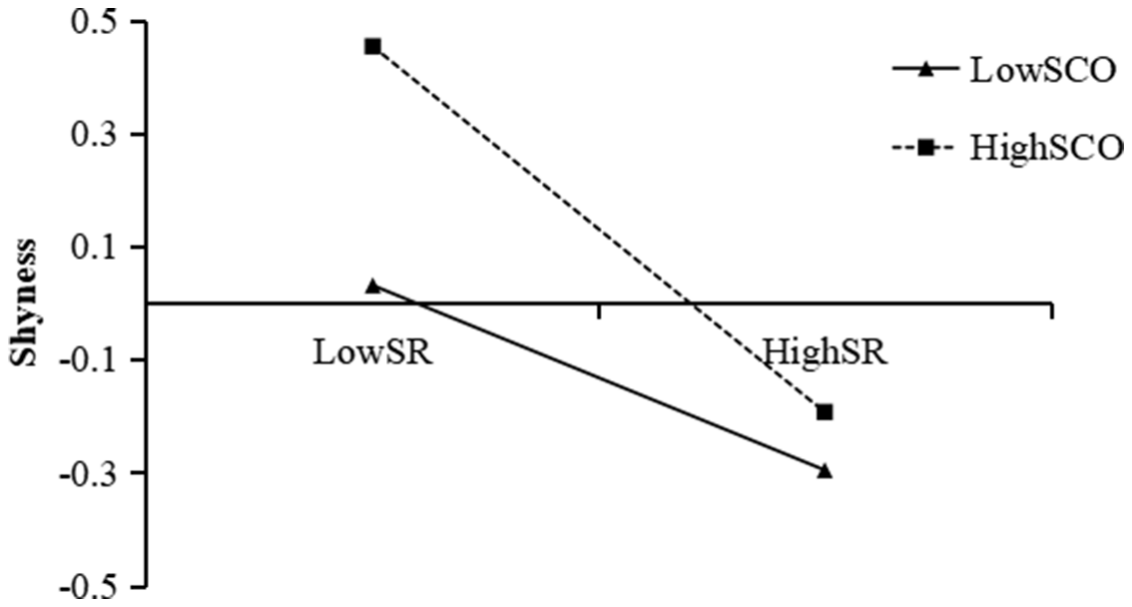
SCO moderated the relationship between SR and shyness. SR, self-regulation; SCO, social comparison orientation.

A simple slope test was also performed to examine the interaction effect of self-regulation and social comparison orientation on shyness. The effect values of self-regulation on shyness were calculated when social comparison orientation was at the mean ± 1SD, and a simple effect analysis graph was plotted. As illustrated in Figure 3, the negative correlation between self-regulation and shyness was significantly higher in adolescents with high social comparison orientation (*β* = −0.337, *t* = −8.308, 95% CI = [−0.416, −0.257]) compared to those with low social comparison orientation (*β* = −0.178, *t* = −4.898, 95% CI = [−0.249, −0.106]).

## 4 Discussion

PIU has widespread adverse effects on individual health, family units, and social development. As Aboujaoude (2017) highlighted in his review, the potential negative impacts of internet-related technologies on personality and their persistence are key issues in internet psychology, warranting further investigation. This indicates that our understanding of how the internet shapes human behavior and social relationships still requires more scientific data and in-depth analysis. This study constructed a moderated mediation model to explore how PIU predicts shyness, the mediating role of self-regulation, and the moderating role of social comparison orientation. The results revealed the complex pathways through which PIU predicted shyness, providing scientific evidence for the formulation and implementation of intervention strategies. These findings not only enriched the theoretical framework of internet psychology but also offered valuable guidance for related practices.

### 4.1 The mediating role of self-regulation

The findings of this study aligned with existing research (LaRose et al., 2003; Yu et al., 2019), indicating a close association between PIU and shyness. Furthermore, this study found that PIU could serve as an effective predictor of shyness. This finding confirmed the profound impact of PIU on offline personality traits, supporting the arguments of Aboujaoude (2017) and Orchard and Fullwood (2010). Additionally, this influence can be explained through the mechanism of self-regulation: there was a significant correlation between PIU and decreased self-regulation ability, which in turn exacerbated individual shyness.

On one hand, PIU may interfere with an individual’s autonomy, particularly in time management and prioritization, thereby impairing self-regulation capacity:

Cognitively, PIU predicted self-regulation capacity in several ways. First, prolonged online activity led to attention fragmentation, directly impacting cognitive control, particularly in sustained attention and task-switching abilities (Fu et al., 2018). This excessive consumption of cognitive resources reduced the efficiency and effectiveness of handling daily tasks, lowering decision-making quality. Second, frequent multitasking, especially switching between multiple windows or devices, may have further impaired executive functions, which are crucial for planning, prioritizing, and inhibiting inappropriate responses (Mostofsky and Simmonds, 2008; Diamond, 2013). Therefore, PIU diminished the ability to maintain goal-directed behavior in complex situations.

Emotionally, PIU predicted self-regulation mechanisms primarily by interfering with emotional processing. Prolonged internet dependency led to difficulties in emotion recognition and expression (Ünal-Aydın et al., 2020), as the emotional feedback provided by virtual environments was often instant and simplified, limiting individuals’ ability to understand and regulate complex emotions. Additionally, PIU can cause emotional dependence, where individuals escaped to the online space to alleviate negative emotions (e.g., loneliness, stress), reducing opportunities to address emotional distress in real life. This weakened the ability to effectively cope with emotional challenges in the real world, which was a critical component of self-regulation (Ünal-Aydın et al., 2020; Cameron and Jago, 2008).

On the other hand, weakened self-regulation may make it more difficult for adolescents to cope with social situations, leading to increased shyness. This phenomenon can be explained through the following psychosocial mechanisms:

First, self-regulation was critical for managing and adjusting behaviors to meet social environment demands. Weakened self-regulation meant reduced ability to control impulsive behaviors, manage emotional reactions, and maintain socially adaptive behaviors. For example, during social interactions, lack of effective self-regulation might prevent individuals from appropriately expressing their thoughts and feelings or remaining calm and focused in stressful and tense situations (Cameron and Nicholls, 1998).

Second, weakened self-regulation also affected social motivation and behavior (Rudolph et al., 2013). When adolescents felt difficulties in controlling their behavior and emotions, they may choose to avoid social situations that seemed to require high self-control. This avoidance behavior not only limited the development of their social skills but also exacerbated their fear and shyness toward social activities.

In summary, these findings emphasized the importance of balancing and integrating online and offline life, as well as the necessity of cultivating self-regulation skills in the treatment and prevention of PIU.

### 4.2 The moderating role of social comparison orientation

This study found that social comparison orientation can mitigate the negative predictive effect of PIU on self-regulation. According to Social Cognitive Theory (Bandura, 1991), individuals can significantly influence their behavior choices by observing the actions of others and their outcomes. In this study, adolescents with high social comparison orientation and PIU were able to better understand and manage their behavior by observing the actions and outcomes of others, thereby enhancing their self-regulation ability. This result was consistent with other findings in the field (Bosch et al., 2010; McIntyre and Eisenstadt, 2011; Stapel and Tesser, 2001). Higher social comparison orientation may mitigate the negative prediction of PIU on self-regulation through three mechanisms: Firstly, by observing and mimicking others’ successful experiences, adolescents can boost their confidence in their abilities, thus more effectively managing and controlling their behavior (Suls et al., 2002; Zell and Alicke, 2009). Secondly, by frequently comparing themselves with others, individuals may reassess the consequences and significance of PIU (Kim et al., 2017), realizing that long-term PIU can negatively impact their life, studies, and future. This understanding may lead them to re-evaluate their behavior and strengthen their willingness and actions for self-regulation. Thirdly, in China, a culture that emphasizes collectivism and social harmony, individuals tend to seek group recognition and social support through social comparison (Markus and Kitayama, 1991). Therefore, adolescents with higher social comparison orientation may pay more attention to the coping strategies and behaviors of their peers and parents, gaining specific behavioral advice and emotional support. This can alleviate the negative emotions and behavioral consequences of PIU, reducing its damage to self-regulation ability. In summary, adolescents with high social comparison orientation can better understand social norms and expectations, adjusting their behavior to conform to these norms, thereby reducing the negative impact of PIU on self-regulation.

Although social comparison orientation can mitigate the negative impact of PIU on self-regulation, it exhibited the opposite effect in the relationship between self-regulation and shyness. This study found that individuals with higher social comparison orientation experience increased shyness when their self-regulation ability is inadequate. This finding revealed the complex mechanisms of social comparison orientation in different psychological phenomena. This phenomenon may be due to individuals with high social comparison orientation more frequently evaluating their relative status and performance compared to others in social environments. This continuous focus on external evaluation can lead to increased stress and anxiety in social situations, especially when individuals feel they cannot meet the standards or expectations of others (Vogel et al., 2015). The persistence of this emotional state places an additional burden on individuals’ self-regulation abilities, as they need to invest more effort to cope with internal discomfort and inadequacy. When the demand for emotional regulation meets already impaired self-regulation abilities, individuals find it more challenging to effectively handle stress in social interactions, thereby increasing their shyness. Additionally, from a physiological perspective, frequent social comparisons and the resulting negative emotions (e.g., jealousy, frustration) may trigger stress responses in individuals. Prolonged stress responses can negatively impact individuals’ mental and physical health, such as increasing cortisol levels, leading to emotional fluctuations and impaired cognitive function (Boehm and Kubzansky, 2012). This physiological burden further weakens individuals’ self-regulation abilities, making it more difficult to cope with stress and challenges in social situations, thus increasing the likelihood of shyness. Finally, adolescence is a critical period for the development of self-concept and social skills. Frequent social comparisons may cause adolescents to focus excessively on external evaluations, neglecting the development of intrinsic values and personal interests (Burnell et al., 2020). This over-reliance on external evaluation and self-inconsistency can lead to a lack of confidence in adolescents when facing social challenges, thereby increasing their shyness levels.

The above research findings indicated that social comparison orientation could enhance the self-regulation abilities of adolescents with PIU, but it had potential negative effects in the relationship between promoting self-regulation and reducing shyness. Specifically, social comparison orientation at different levels affected the relationship between PIU and shyness prediction through self-regulation differently. When social comparison orientation was high, the negative impact of PIU on self-regulation was alleviated, but the negative impact of self-regulation on shyness was intensified. With a high level of social comparison orientation, individuals might have enhanced their self-regulation abilities by observing others’ behaviors and outcomes, thereby reducing the negative impact of PIU on self-regulation. However, this high social comparison orientation also made individuals more susceptible to feeling pressure and expectations from others when facing insufficient self-regulation, thus exacerbating shyness. Conversely, when social comparison orientation was low, individuals might have been less influenced by others’ behaviors, had relatively weaker self-regulation abilities, and experienced a greater negative impact of PIU on self-regulation. However, due to fewer external comparisons, shyness was relatively low. This indicates that the mechanism by which PIU predicts shyness through self-regulation varies with different levels of social comparison orientation.

The reason for the opposite effects of social comparison orientation in the mediation model might be due to the Iowa–Netherlands Comparison Orientation Measure (INCOM) used in this study, which only distinguished the level of social comparison orientation and could not effectively differentiate its direction. However, existing research showed that upward comparison and downward comparison have different effects on individuals’ psychology and behavior. For example, Buunk et al. (2005) found that students often experienced stronger negative emotions, such as inferiority and anxiety, when engaging in upward comparison in the classroom. In contrast, downward comparison could make adolescents feel proud and stimulate their motivation for self-improvement (Gürel et al., 2022). In the first part of the pathway in this study, downward comparison orientation might have helped alleviate the negative impact of PIU on self-regulation, while in the second part of the pathway, upward comparison orientation might have exacerbated the prediction of shyness by self-regulation.

In summary, the moderating effect of social comparison orientation in the mediation model exhibited a “double-edged sword effect,” indicating that the pros and cons need to be weighed and its complexity fully recognized when using social comparison orientation as an intervention measure. We need to seek an optimal level of social comparison orientation that can maximize the alleviation of the negative prediction of PIU on self-regulation while minimizing the exacerbation of shyness by self-regulation, ultimately reducing the negative prediction of PIU on shyness.

### 4.3 Limitations and implications

This study had several limitations that need to be addressed in future research. Firstly, this study used a cross-sectional design, which cannot infer causality between PIU and shyness. Future research should use experimental and longitudinal designs to further verify this association. Secondly, the measurement of social comparison orientation in this study was inadequate. Future research should use more comprehensive measurement tools to accurately assess different types and directions of social comparison orientation (e.g., upward and downward social comparison orientation). This will help explore their roles in different pathways of the mediation model, providing a more comprehensive understanding of the complex relationships between PIU, self-regulation, and shyness, and offering a more scientific basis for designing intervention measures. Thirdly, as a global phenomenon, esports not only attracts a large number of players and spectators but has also been included as an official event in the 2022 Asian Games and the 2024 Olympic Games. Current research has not adequately distinguished between professional esports and PIU. The extensive training required for professional players is mistakenly viewed as addictive behavior, whereas it is actually a professional necessity (Griffiths, 2017). Future research should focus on differentiating between professional gaming participation and gaming addiction behavior, and explore its impact on self-regulation abilities, to maintain competitive levels while protecting the mental and physical health of players (Bányai et al., 2019).

The results of this study have important implications for educational practice. Firstly, educators can gain a deeper understanding of the potential dangers and complex impacts of PIU on adolescent mental health, particularly its predictive role in shyness personality traits. This will help educators take PIU into account when identifying and understanding student behavior and emotional problems. At the same time, attention should be paid to adolescents’ internet use habits in daily life, with timely detection and intervention of potential PIU behaviors. Educators should actively participate in supervising adolescents’ internet use and help them establish healthy internet habits. Secondly, the importance of adolescents’ self-regulation abilities should be emphasized. This includes helping students learn key skills such as time management, priority setting, and emotional regulation. Thirdly, attention should be paid to the double-edged sword effect of social comparison orientation. Educators should guide adolescents to engage in positive social comparison to stimulate their motivation for self-improvement while avoiding excessive negative comparisons to reduce anxiety and inferiority feelings. Finally, as esports is an emerging trend, educators should keep pace with the times, understand and apply the latest research findings in this field. This includes distinguishing between professional gaming participation and pathological gaming addiction to avoid misjudgments and inappropriate interventions. Educators should also collaborate with experts in related fields to study and explore the impact of esports on self-regulation and develop scientific education and training programs.

## Data Availability

The original contributions presented in the study are included in the article/supplementary material, further inquiries can be directed to the corresponding author.
